# Influence of Fuel Level on Properties, Productivity, and Mineralogy of Russian Vanadiferous Titanomagnetite Sinter

**DOI:** 10.3390/ma14216258

**Published:** 2021-10-21

**Authors:** Jiahao Li, Jingwei Men, Songtao Yang, Mi Zhou

**Affiliations:** 1 School of Materials and Metallurgy, University of Science and Technology Liaoning, Anshan 114051, China; lijiahao923@163.com (J.L.); menjingwei923@163.com (J.M.); 2 Key Laboratory of Liaoning Province for Recycling Science of Metallurgical Resources, Northeastern University, Shenyang 110819, China; 3 School of Metallurgy, Northeastern University, Shenyang 110819, China

**Keywords:** sinter, mineralogical phases, metallurgical properties, blast furnace

## Abstract

The influence of fuel level on Russian vanadiferous titanomagnetite sinter properties, productivity, and mineralogy are researched by sintering pot testing, metallographic microscopy, scanning electron microscopy analysis, and energy dispersive spectrometer (SEM-EDS) analysis. A comprehensive index is evaluated in conjunction with the same indexes and significance coefficient as that in the Panzhihua Iron and Steel Group. Results show that with the increasing fuel level from 3.5% to 6.0%, flame front speed, yield, tumbling test index (TI), and productivity, all first increase and then decrease. The low temperature reduction degradation index (RDI_+3.15_) and softening zone (ΔT) gradually increase while the RI and starting temperature of softening (T_10_), and ending temperature of softening (T_40_) decrease with increasing fuel levels from 3.5% to 6.0%. With the increase of fuel level from 3.5% to 6.0%, the content of FeO, SiO_2_, and MgO increase, while TiO_2_ shows a decrease. For the same increase in fuel level, the number of pores and calcium ferrite and hematite decrease but the silicate increases. In addition, in the fuel level range of 3.5% to 5.5%, magnetite correspondingly increases but then shows a drop after 5.5%. Moreover, when the fuel level increases to greater than 5.0%, FeOx and fayalite quickly increase and a small amount of metallic iron appears under the fuel level of 6.0%. Overall, the optimal fuel level under current production conditions and indicator selection is 4.0%.

## 1. Introduction

Russian vanadiferous titanomagnetite (RVT) ore has a high utilization value due to its iron, vanadium, titanium and chromium element. Currently, the mainstream route of the utilization of RVT ore is the blast furnace (BF)→basic oxygen furnace (BOF) [1,2]. As an important blast furnace charge, the properties of RVT sinter have important impacts on blast furnace smelting while the productivity affects production cost. Due to the complex compositions of minerals, chromium spinel (FeO·Cr_2_O_3_) with a high melting point (1850 °C) causing the assimilation temperature to be too high (1335 °C) [2,3], and the insufficient liquid phase volume and perovskite level, the properties, and productivity of RVT sinter are unsatisfactory.

Extensive work on vanadiferous titanomagnetite properties and productivity has been carried out to obtain higher properties and high productivity with respect to production [4,5,6,7,8,9,10,11]. Separately, extensive work has been performed to improve the sinter mineral composition and structure, particularly the sinter bonding phase using analytical reagent, to simulate the actual sinter with a specific focus on the effect of chemical composition on mineral composition and structure [12,13,14,15,16,17,18,19,20,21,22]. In the sintering process and beyond the chemical composition (MgO, TiO_2_, CaO, SiO_2_, B_2_O_3_, etc.), temperature and atmosphere have an important impact on the properties, productivity, and mineralogy of sinter. It is well known that a heat source is supplied by fuel combustion to satisfy the energy needs for mineralization reactions in the sintering process of iron ores, but this process also releases heat and produces necessary atmosphere for the production of high-basicity sinter, such as carbon monoxide, that can disturb the formation of calcium ferrite. Zhang, J. et al. reported the effect of temperature and atmosphere on calcium ferrite by analytical reagent [23]. Webster and N.A.S. et al. reported the effects of oxygen partial pressure on the formation mechanism of calcium ferrite in the sintering process [24]. Wang and W.S. et al. reported the effects of coke content on the properties of V-Ti by sintering pot tests [25], while Fan and X.H. et al. researched the influence of O_2_ concentration in the circulation gas on the microscopic structure of the sinter [26]. However, the properties, productivity, and mineralogy were never considered simultaneously and in conjunction with production conditions in any research.

To generate better utilization of RVT ore, their properties, productivity, and mineralogy must be considered together and in conjunction with production conditions. In this context, the influence of fuel level on RVT sinter properties (tumbling test index, size distribution, low temperature reduction degradation, reducibility, and softening properties), productivity (flame front speed and yield), and mineralogy were researched by sintering pot tests, metallographic microscope, scanning electron microscope analysis, and energy dispersive spectrometer analysis (SEM-EDS). The comprehensive index was evaluated through the comprehensive index method [12], combined with the same indexes and significance coefficient as that in the Panzhihua Iron and Steel Group (PANGGANG—the largest vanadium titanium smelting company in the world [27]), to help optimize fuel level under current production conditions and indicators.

## 2. Materials and Methods

### 2.1. Raw Materials

**Figure 1:**
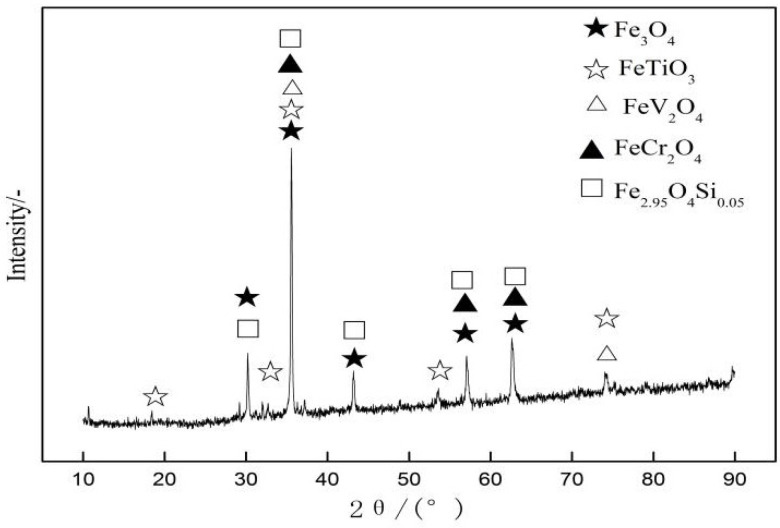
XRD pattern of RVT ore.

**Figure 2:**
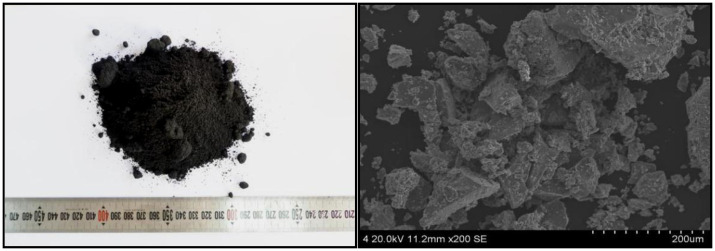
Macro- and micro- structure of RVT ores.

RVT ore was supplied by ARICOM Group Company, Blagoveshchensk, Russia, and the other raw materials were supplied by Jianlong Iron and Steel Group Company, Shuangyashan, China. The chemical composition of the raw materials and coke breeze for experimental work are listed in Table 1 and Table 2. The RVT ore has a total iron (TFe) content of 61.54%, a TiO_2_ content of 5.17%, and lower SiO_2_ and MgO contents of 2.37% and 1.21%, respectively. The XRD pattern of RVT magnetite is shown in Figure 1. RVT ore mainly consists of magnetite (Fe_3_O_4_/Fe_2.95_O_4_Si_0.05_), ilmenite (FeTiO_3_), vanadium spinel (FeO·V_2_O_3_), and chromium spinel (FeO·Cr_2_O_3_). As seen in Figure 2, the RVT ore is black brown, and its edge angle is smooth and vertical. The structure is dense and there are not many fine particles and pores, so it can be concluded that the granulation is poor.

### 2.2. Experimental Procedure

#### 2.2.1. Schemes of Sintering Pot Tests at Different Fuel Levels

A 13% RVT magnetite ore was added in the sinter mixture, and the sinter mixture was calculated according to the target sinter chemical composition in Table 1 and Table 2. The mass fraction of magnetite A is ~15%, magnetite B is ~20%, magnetite C is ~12%, shaft furnace dust is ~4.5%, return fines is ~14%, and magnesite is ~3.0%. The basicity (R = CaO/SiO_2_) was maintained at 2.25, and fuel levels (coke level) were 3.5%, 4.0%, 4.5%, 5.0%, 5.5%, and 6.0%, respectively.

#### 2.2.2. Sintering Pot Tests

The process of the sinter pot test consists of the steps of sinter proporting, mixing, granulation, loading into pot, ignition, sintering, cooling, breakering, dropping, screening, and product sinter, in that order [16]. The experimental raw materials were sintered and dosed in the proportions described in Section 2.2.1, then mixed well. Then, the sinter blend mixture used tice-mixed granulation, and the sinter moisture was 7.5 ± 0.3%. The granule sinter mixture was charged into a sintering pot of 150 mm diameter, and the bed height was maintained at 500 mm. The hearth layer was 20 mm and comprised of 10–15 mm sinter. Ignition is carried out at a temperature of 1000 °C and a negative pressure of 5 kPa for 2 min. During the sintering process the negative pressure is 10 kPa and the sintering end point is reached when the sintered exhaust gas temperature reaches its maximum. The sintering test parameters, which were kept fixed, are listed in Table 3.

The cooled sinter cake was breakered with a sinter breaker (Lichen Technology Company, Shanghai, China) and then dropped from a height of 2 m three times. The sinter was then screened into five sizes of fractions: >40 mm, 25–40 mm, 10–25 mm, 5–10 mm, and <5 mm. A mixture of 25–40 mm, 10–25 mm and 5–10 mm sinter of similar particle size distribution was taken and then crushed into 10–12.5 mm sinter for further metallurgical property evaluations and microscopic testing analysis.

#### 2.2.3. Mineralogical Investigations

**Figure 3:**
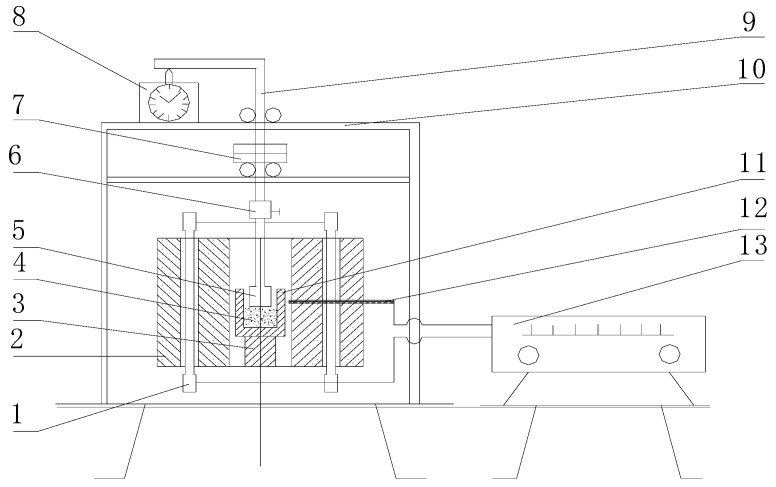
Schematic diagram of the experimental apparatus: 1, Si-C heater; 2, shell; 3, Al_2_O_3_ Pedestal; 4, sample; 5, Si-C bar; 6, fastening screws; 7, load; 8, m 9, steel bar; 10, bracket; 11, graphite crucible; 12, thermocouple; 13, temperature program controller).

The metallurgical properties of sinter, including the tumbling test index (TI), low temperature reduction degradation index (RDI), and reducibility index (RI), were determined according to ISO-3271, ISO-4696, and ISO-7215, respectively. Sinter is not a pure crystalline substance and is without a fixed melting point; it is just within a certain zone of soft melting. In blast furnace production, a higher melting temperature and narrower softening zone are better to stabilize the gas–solid phase operation and movement of the blast furnace gas. In this experiment, T_10_ defines the starting temperature of softening with the height of the material layer with 10% shrinkage, T_40_ defines the ending temperature of softening with the height of the material layer with 40% shrinkage, and ΔT = T_40_-_10_ defines the softening zone. The particle size of the sinter samples was 1.5~2.5 mm, the load was 1 kg/cm^2^, and the height of the material layer was 40 mm. The schematic diagram of the experimental apparatus is shown in Figure 3 [15].

The sinter samples obtained from each experiment were mounted in epoxy resin, polished after treating in coarse sandpaper and smooth frosted glass panel, and characterized by an optical microscope (DM1750M, Leica Microsystems, Wetzlar, Germany). Mineral phase microscope analysis data gave the average statistical area of each mineral phase, while the total observational scope area was characterized with an image analyzer (Cambridge Q500, Leica Microsystems, Wetzlar, Germany) and SEM-EDS (S-3400N, JEOL Ltd., Tokyo, Japan).

## 3. Results

### 3.1. Influence of Fuel Level on Particle Size Distribution

**Figure 4:**
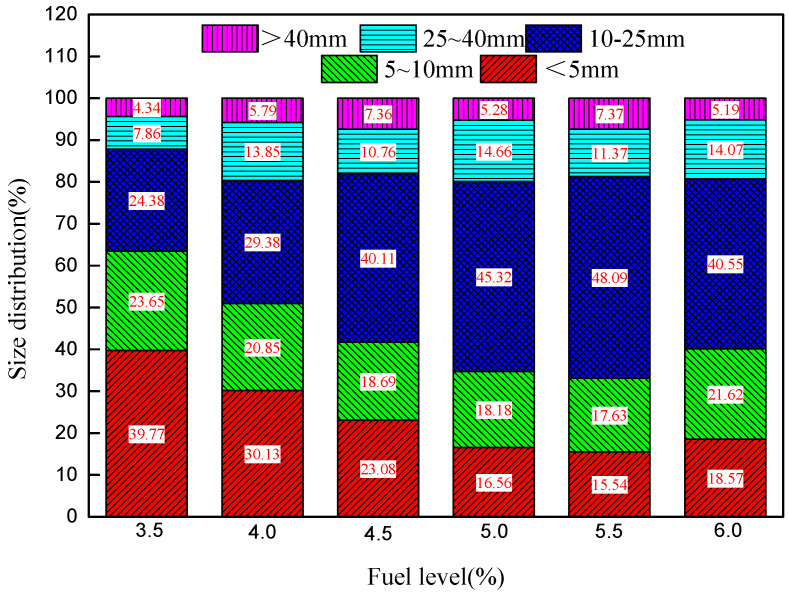
Effect of fuel content on particle size distribution of RVT sinter.

The chemical compositions of RVT sinters (+5 mm) containing different fuel levels are shown in Table 4. As shown in Table 4, the content of FeO rapidly increases with increasing fuel level (3.5–6.0%) and SiO_2_ content increases from 5.37% to 5.81%. The reasons for this and the effect on metallurgical properties will be discussed in Section 4. Figure 4 reports the particle size distribution of the sinter after falling. As shown, with increases in fuel level from 3.5% to 5.5%, the −10 mm particle size percentage decreases from 63.42% to 33.17%, the 10–25 mm size percentage increases from 24.38% to 48.09%, and the +40 mm size percentage increases. When the fuel level increases further to 6.0%, the −10 mm size percentage increases and the 10–25 mm size decreases, while the +25 mm size remains the same. The size distribution mainly depends on the liquid volume and the type of bonding phase; 5.0% is best just in the sight of size distribution.

### 3.2. Influence of Fuel Level on Mineralogy and Microstructure

**Figure 5:**
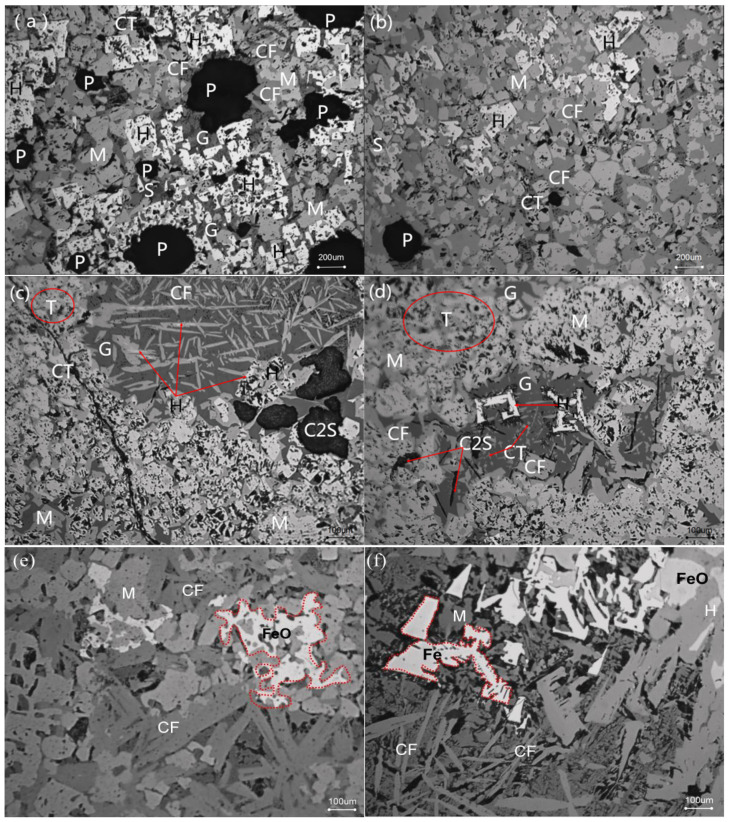
Mineralogy and microstructure of RVT sinter at different fuel levels (H: hematite, M: magnetite, CF: calcium ferrite, S: silicate, G: glass, CT: perovskite, P: pore, C2S: dicalcium silicate, FS: fayalite, T: over-melt zone): (**a**) Fuel-3.5%, (**b**) Fuel-4.0%, (**c**) Fuel-4.5%, (**d**) Fuel-5.0%, (**e**) Fuel-5.5%, (**f**) Fuel-6.0%.

**Figure 6:**
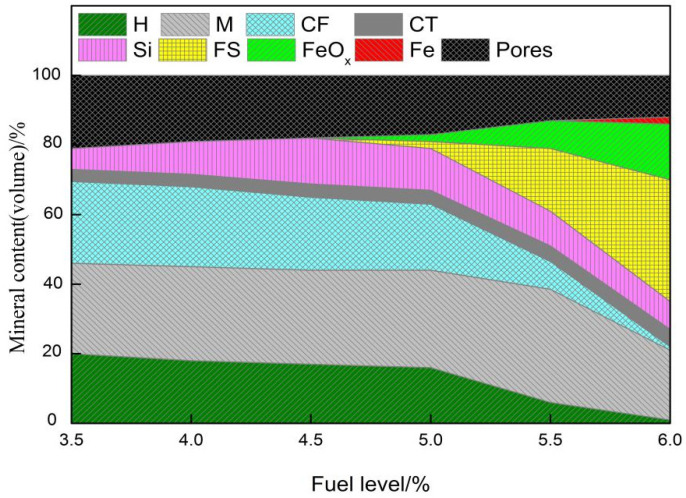
Mineralogy variation of RVT sinter at different fuel levels (H: hematite, M: magnetite, CF: calcium ferrite, Si: silicate (dicalcium silicate, glass, etc), CT: perovskite, FS: fayalite).

Effects of fuel level on mineralogy and microstructure are shown in Figure 5 and Figure 6. As seen from Figure 5 and Figure 6, with increasing fuel level, there is a large variation in the mineral composition, structure, and morphology of RVT sinter. In the sample with 3.5% fuel, a large number of hematite and magnetite phases are present in the surrounding pores. In addition, magnetite and hematite are bonded by calcium ferrite, silicate, and a small amount of glass (Figure 5a). As the fuel level increased from 3.5% to 4.0%, the sinter microstructure remains uniform, and the number of pores and amount of hematite decrease. Moreover, a large amount of magnetite bonded by calcium ferrite, a small amount of silicate, and perovskite, appear and become distributed in the regions between magnetite, calcium ferrite, and the surroundings around the pores (Figure 5b). When the fuel level increases to 4.5%, the higher liquid volume leads to fewer pores, but the degree of increase in the liquid volume is not too great. Another finding is the appearance of secondary hematite through the decomposition of calcium ferrite (Figure 5c). Calcium ferrite decreases while silicate (dicalcium silicate and vitreous) and perovskite increase. In addition, there are cracks throughout the region where perovskite precipitates. Furthermore, another finding is the phenomenon that some nuclear particles become over-melted in some regions (Figure 5c). When the fuel level further increases to 5.0%, more liquid, perovskite, and dicalcium silicate are generated, while the number of pores and hematite content further decrease. In addition, the over-melting of nuclear particles further intensifies (Figure 5d), and more secondary hematite and perovskite precipitate from the calcium ferrite (Figure 5d) and silicate phases (Figure 5d), respectively. As the fuel level rises to 5.5%, a large number of nuclear particles are in the molten state and do not hold the original morphology of the grain. Meanwhile, a large number of FeOx and fayalite (calcium and iron olivine) appear but the majority of calcium ferrite has decomposed and decreased rapidly, where a similar trend is observed in hematite and number of pores (Figure 5e). As the fuel level increases to 6.0%, another interesting finding is as follows: a small amount of metallic iron appears in the highly reduced region (Figure 5f). The microstructure of the sinter presents a molten state with low porosity, where the main minerals consist of FeOx, fayalite (calcium and iron olivine), dicalcium silicate, magnetite, silicate, and perovskite with little calcium ferrite and hematite.

### 3.3. Operation Parameters of Sintering Process

**Figure 7:**
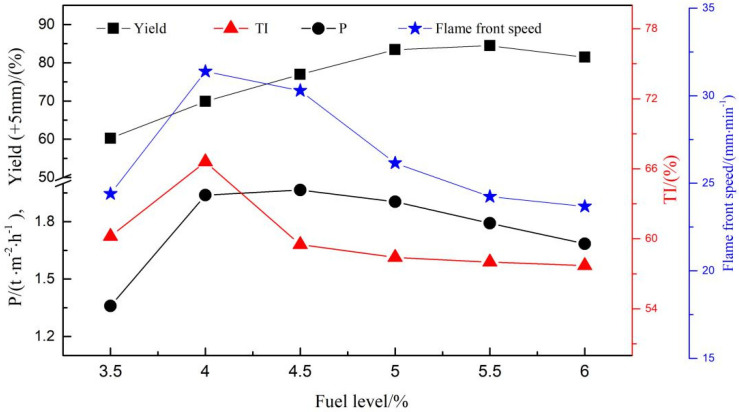
Effect of fuel content on the metallurgical properties and productivity of RVT magnetite sinter.

Figure 7 shows the variation of flame front speed with respect to fuel level. As seen in Figure 7, when the fuel level increases from 3.5% to 4.0%, the flame front speed increases from 24.39 to 31.38 mm·min^−1^. As the fuel level extends from 4.0% to 6.0%, the flame front speed decreases from 31.38 to 23.67 mm·min^−1^.

Figure 7 also reports the variation of yield with respect to fuel level. It shows that the yield increases from 60.23% to 84.46% when the fuel level gradually increases in its range from 3.5% to 5.5%, while there is a drop in yield (84.46% to 81.43%) when the fuel level increases from 5.5% to 6.0%

It can be easily found from Figure 7 that TI rapidly grows from 60.2% to 66.6% with a 6.4% growth as the fuel level increases from 3.5% to 4.0%, but as the fuel level rises to 4.5%, TI rapidly decreases to 59.5% and to 57.7% when the fuel level further increases to 6.0%.

Figure 7 also shows the effect of fuel level on productivity. It can be seen in the figure that as the fuel level increases to 4.0%, there is a huge increase in productivity from 1.359 to 1.939 t/(m^2^·h) and rises to 1.965 t/(m^2^·h) when the fuel level further increases to 4.5%, but when the fuel level increases to higher levels, there is a reduction in productivity. The productivity is calculated as shown in Equation (1).

(1) $$P=\frac{\mathrm{Ms}*y}{A*t}$$

where *P* is the productivity (t/(m^2^·h)), M_S_ is the mass of sinter cake (t), y is the yield of product sinter (%), A is the cross sectional area of the sintering pot (m^2^), and t is the sinter time (h).

As seen in Figure 7, in the range of fuel level from 3.5% to 4.0%, flame front speed increases and sintering time t reduces. Meanwhile, y shows an increase, and *p* exhibits a huge overall increase. When the fuel level increases to 4.5%, the flame front speed decreases with an increase in t, but at the same time y has a further increase and the rate of y increasing is larger than that of t, therefore, *p* shows an even higher increase. When the fuel level increases to 5.0%, though y and t have increased, the rate of t increasing is larger than that of y increasing, therefore, *p* decreases. *p* shows a huge decrease for the decrease of y and increase of t when the fuel level extends from 5.5% to 6.0%.

### 3.4. Effects of Fuel Level Onmetallurgical Properties

**Figure 8:**
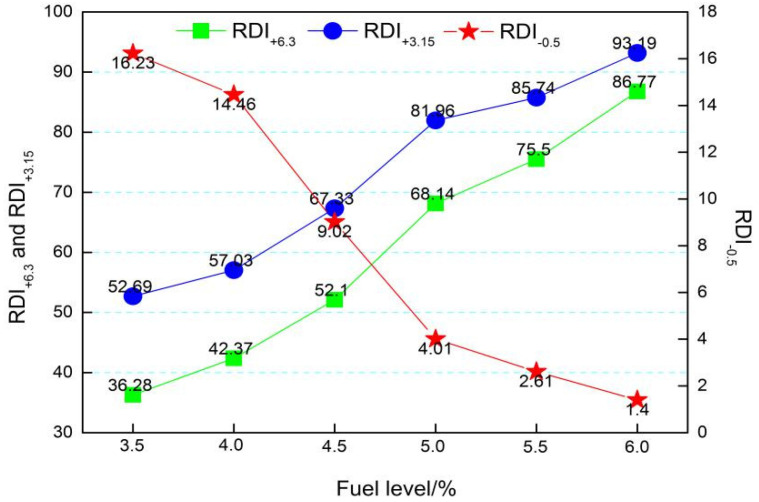
Effect of fuel level on the RDI of RVT sinter.

**Figure 9:**
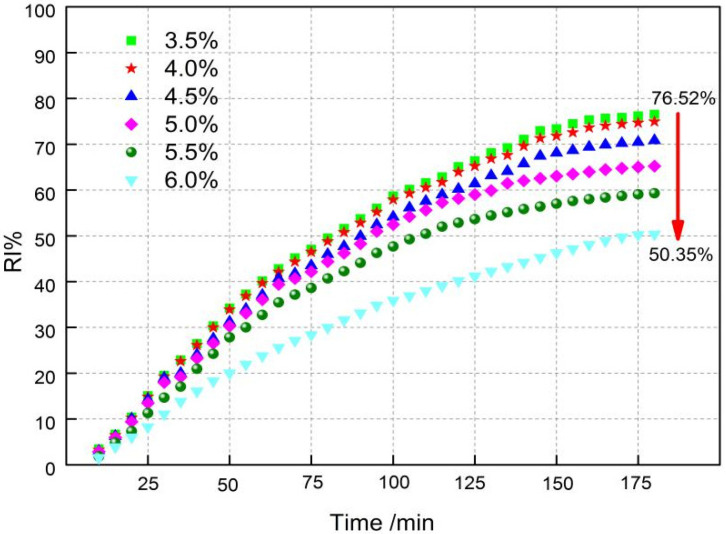
Effect of fuel content on the RI of RVT sinter.

The RDI is a measure of the disintegration susceptibility of the sinter under exposure to CO in the temperature range of 450 °C to 600 °C. The RDI of the sinters was determined by the ISO4696 test procedure of static reduction and followed by tumbling. This test simulates the blast furnace conditions in the upper stack regions with mild reducing conditions and relatively low temperatures. After the reduction following tumbling, the +3.15 mm fraction generated is considered as the RDI (+3.15 mm) of the sinter, where an RDI (+3.15 mm) greater than 70% is considered a good RDI. RDI_+3.15_ is the assessment index, and RDI_+6.3_ and RDI_−0.5_ are reference indexes. It can be seen in Figure 8 that fuel level has a significant impact on RDI. The RDI_+6.3_ and RDI_+3.15_ increase from 36.28% and 52.69% to 86.77% and 93.19%, respectively, with a reduction of RDI_−0.5_ value from 16.23% to 1.40% when the fuel level gradually increases from 3.5% to 6.0%. As shown from Figure 9, RI reduces from 76.52% to 50.35% with increasing fuel level.

**Figure 10:**
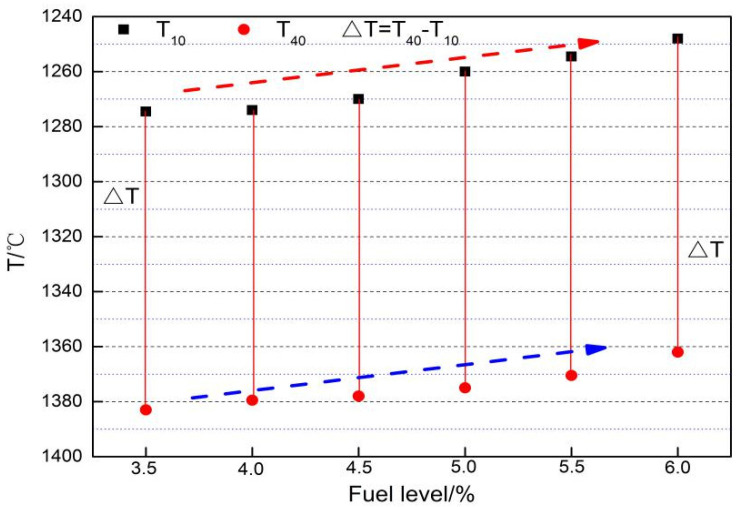
Effect of fuel content on the softening properties of RVT sinter.

Figure 10 reports the variation of T_10_, T_40__,_ and ΔT with increasing fuel level. It is found that with an increase of fuel level from 3.5% to 6.0%, T_10_ andT_40_ decrease from 1274.5 °C, and 1383 °C to 1248 °C and 1362 °C, respectively. Also, ΔT expands from 108.5 °C to 114 °C with a widened softening zone. It is not beneficial for the movement of blast furnace gas and the stability of a gas–solid phase operation.

### 3.5. Comprehensive Index

Since the corresponding fuel level is not necessarily the same when each of the indexes obtain their optimal performance, the fuel level that obtains one of the best indexes may not be the best overall fuel level. Indicators under different fuel levels are needed for a comprehensive study. Therefore, we use the comprehensive index method, which is widely used at home and abroad, to help us search for the optimal fuel level. The indicators that the higher the better are defined as positive indicators, such as productivity, RDI_+3.15_, and RI, while the indicators that the lower the better are defined as negative indicators, such as fuel level. In this FFpaper, the comprehensive index method is the same as the method in Ref. [12], and the indexes chosen and significance coefficient are the same as that in PANGGANG [27]. The indexes chosen are productivity, TI, RDI_+3.15_, RI, and fuel level. The total significance coefficient is 100, and the significance coefficient of productivity, TI, RDI_+3.15_, RI, and fuel level are 20, 30, 20, 15, and 15, respectively. The comprehensive evaluation index is obtained through Equations (2)–(5), and the calculation process of the comprehensive index is shown in Table 5. The method is as follows.

To facilitate the comparison and analysis, the comprehensive index of the first group is set as a reference and quantified to 100.

Define:

(2) Fi=fi−f1+100

where the comprehensive index is the composite index.

And F1=100 (F1=f1−f1+100)

(3) $$fi=\sum_{j=1}^{m}\omega jzij\;(i=1,2,\ldots n;j=1,2,\ldots m;)$$

where ωj is the unit range coefficient.

And $f_{i}\geq 0\;(i=1,2,\ldots ,n)$

(4) $$\omega j=\frac{Wj}{Rj}$$

where Wj is the significance coefficient and $\sum_{j}Wj=100$ and Rj is range.

(5) $$Rj={\left(zij\right)}_{\max}-{\left(zij\right)}_{\min}$$

where zi_1_ is productivity, zi_2_ is TI, zi_3_ is RDI_+3.15_. zi_4_ is RI, and zi_5_ is fuel level.

It can be seen in Table 5 that as fuel level increases from 3.5% to 6.0%, the comprehensive index shows an initially large increase and then a continuous decrease. The highest comprehensive index is 138.95 at a 4.0% fuel level. Therefore, the optimal fuel level is 4.0% under current production conditions and indicators.

## 4. Discussion

The sintering of iron ore relies on the combustion of fuel added to the mix to provide the heat necessary for its mineralization and to change the atmosphere of the sinter layer. This has a significant impact on the mineralization process of the sinter mix and changes the mineral organization and structure of the sinter, which has a significant impact on the quality of the sinter production.

As seen in Table 4, the FeO content in the sintered ore rapidly increases with increasing fuel levels (3.5−6.0%). The FeO content in the sinter is related to two factors: one is generating content of FeO and the other is the reoxidizing content of FeO. Their relationship is as follows: W_FeO(forming)_ = W_FeO(generating)_ − W_FeO(reoxidizing)_. According to the relationship above, it can be further speculated that the influence of fuel level on sinter is also affected by changing temperature and atmosphere. With fuel level increasing, the temperature increases, which is beneficial in generating more melt with FeO and increases W_FeO(generating)_. At the same time, the oxidizing atmosphere weakens and reducing atmosphere intensifies, which reduces W_FeO(reoxidizing)_. With increasing FeO, more melt with a lower melt point forms for SiO_2_ to easily react with FeO to form 2FeO·SiO_2_. This may indicate the reason that SiO_2_ content rises with increasing fuel level. Generally, MgO content also slightly increases, which is attributable to the formation of MgO·FeO·SiO_2_ with the increasing fuel level. In contrast, TiO_2_ content decreases with the formation CaO·TiO_2_, and with the fuel level increasing, the temperature rises and the reducing atmosphere enhances, which is beneficial to the formation of perovskite (CaO·TiO_2_). It is well known that perovskite is not the bonding phase, and it has high hardness but low compression strength, which is poor for strength and yield. It is one of the reasons that the return fines (−5 mm) of V–Ti sinter are larger than that of the ordinary sinter. In other words, TiO_2_ returned more fines (−5 mm) in the form of perovskite (CaO·TiO_2_). So, with the fuel level increasing, the content of perovskite (CaO·TiO_2_) increases and more TiO_2_ in the form of perovskite (CaO·TiO_2_) enters to return fines (−5 mm); therefore, the TiO_2_ decreases in the sinter (+5 mm).

**Figure 11:**
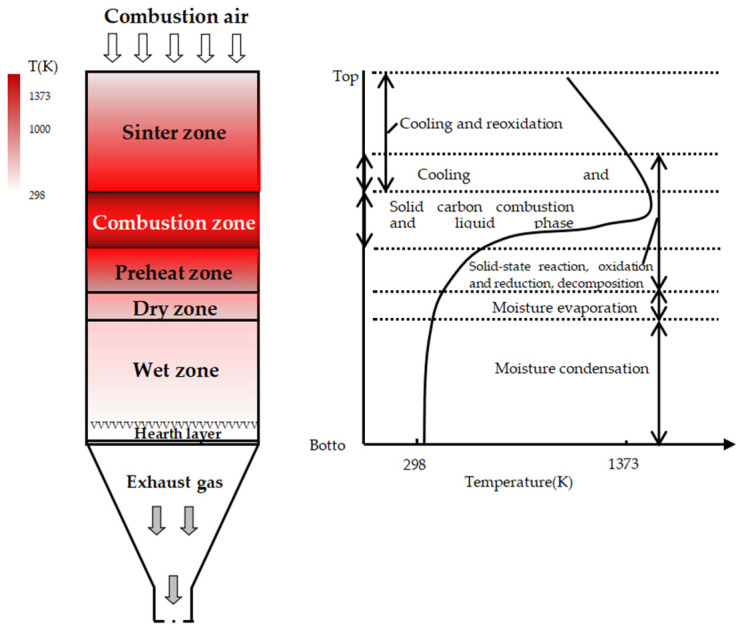
Schematic diagram of the sintering reaction.

As seen from Figure 7, the sintering flame front speed first increases and then decreases. This is probably due to the large heat losses and low heat provided by 3.5% fuel combustion, which resulted in a lower heat that can be used for the mineralization of the sinter mixture and caused the flame front speed to decrease at the 3.5% fuel level. Therefore, with the fuel level increasing to 4.0%, more heat is provided by more fuel combustion, which can provide sufficient heat for the mineralization of the sinter mixture, which increases the flame front speed. It is well known that fuel combustion not only releases heat to improve the temperature but also alters the atmosphere in the sintering mixture. The schematic diagram of the sintering reaction is shown in Figure 11 [28,29]. The fuel is mainly burned in the combustion zone; the sintering mix forms low melting point minerals under the solid phase reaction and softens at a high temperature, with further development producing a liquid phase. The combustion zone has a great impact on the yield and quality of the sinter, where too thick affects the permeability of the material layer, resulting in lower yield, and too thin is a low sintering temperature, the number of liquid phases are insufficient, and the sinter is not well consolidated. With the increase in fuel level from 4.0% to 4.5%, more liquid is generated for more heat released by fuel combustion, which causes a decrease in the permeability of the mixture. Meanwhile, more O_2_ is consumed for fuel combustion and the decrease of air flowing through the mixture decreases the permeability, which causes the decrease of the lower oxygen partial pressure and fuel reactivity. Hence, the flame front speed decreases. Specifically, when the fuel level rises to 6.0%, the amount of the liquid generated is too much due to the temperature being too high for the excessive heat to be released by too much fuel combustion, which causes the voids and porosity to decrease too much. Therefore, the diffusion velocity of the air through the material layer and the O_2_ content decrease quickly, which is one of the reasons for the appearance of unburned fuel, which may deteriorate the flame front speed further to 23.67 mm·min^−1^.

**Figure 12:**
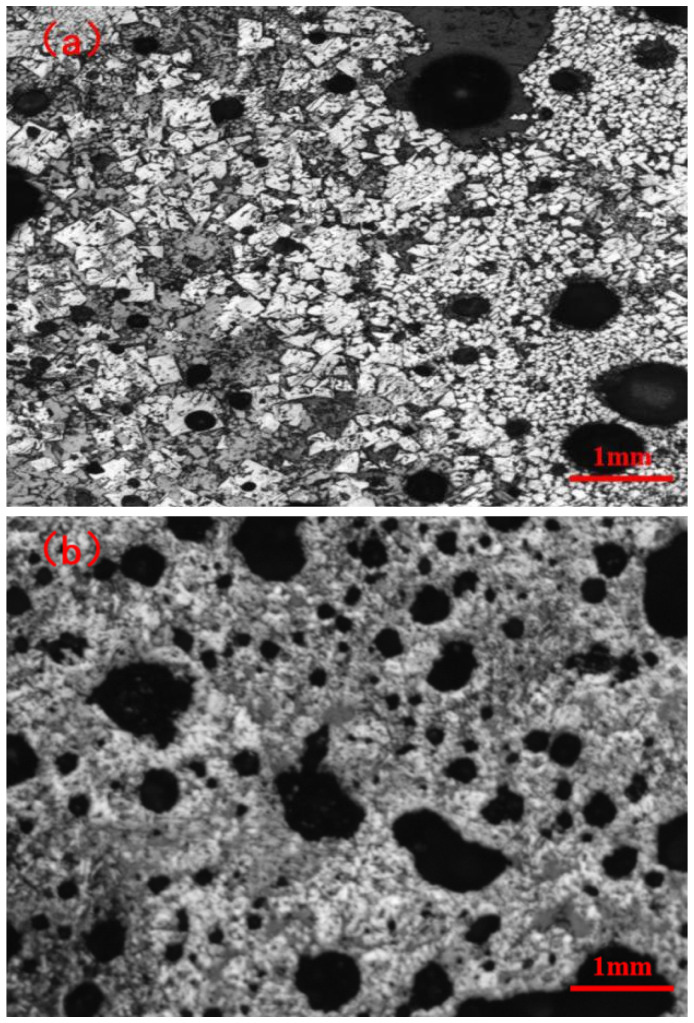
Macrostructure diagrams: (**a**) Thick-walled, small pore structure (Fuel-4.0%), (**b**) Thin-walled, large pore structure (Fuel-6.0%).

In addition, as seen from Figure 7, the yield of sinter follows the same trend as the sintering flame front speed. This may be attributed to the increase in liquid volume and the decrease in pores with the extent of fuel level (3.5% to 5.5%). In addition, because of the decrease in flame front speed, the molten melt has more time to complete crystallization and reduce the vitreous generated (Figure 6). However, the nuclear particles are over-melted with a lower strength for the too high temperature released by the 6.0% fuel combustion, and results in a thin-walled and large-holed macrostructure (Figure 12). Moreover, the increase of perovskite and reduction in calcium ferrite may be the other reasons contributing to the reduction in yield (Figure 6). Overall, the fuel level should not be too high and 5.5% is best with respect to yield.

As seen from Figure 7, the TI of the sintered ore also follows the same trend as the sintering flame front velocity. When the fuel content is low, the heat released by fuel combustion is not enough to generate sufficient liquid volume. Hence, TI was lower for more pores and has less bonding phase at 3.5% fuel level (Figure 5a and Figure 6). Therefore, with the fuel level increased to 4.0%, more liquid volume is generated through sufficient heat released by proper fuel combustion, and more bonding phase and less pores are the reasons that TI shows rapid growth. When the fuel level is increased to 4.5%, there is more heat and liquid, with less pores, flow air, and less O_2_ generated, which causes the sintering mixture layer with a higher temperature and lower oxygen partial pressure. This is not conducive to the production of calcium ferrite, especially the silico-ferrite of calcium and aluminum (SFCA) with high strength, and causes calcium ferrite to decompose to secondary hematite (Figure 5c and Figure 6). Consequently, TI decreases rapidly. With the fuel level increasing to 5.0%, more calcium ferrite decomposes and more perovskite is generated. Moreover, the nuclear particles exhibit a slight over-melt, so TI decreases further. As the fuel level increases to 6.0%, the nuclear particles in the over-melt show lower strength and result in a thin-walled, large holed macrostructure (Figure 12). Therefore, TI has a further decrease through more liquid and less pores (Figure 5d,e and Figure 6).

Figure 8 and Figure 9 show that the RDI of sintered ore improves and the RI decreases as the fuel level increases. It is known that the fundamental reason for the weakening and degradation of sinter is the phase transformation of Fe_2_O_3_→Fe_3_O_4_, which is associated with a volume increase of 10% and leads to powder during the reduction process at 450~500 °C. It can be easily found from Figure 5 and Figure 6 with the fuel level improving that the volume fraction of Fe_2_O_3_ has a fast decrease, which may be the main reason that RDI has a large improvement. In addition, the increase of FeO (Table 4) and decrease in pores (Figure 5 and Figure 6) are beneficial to the improvement of RDI. A 1% increase in FeO improves RDI by four points [30]. Furthermore, with more precipitation of Mg-based olivines (MgO·FeO·SiO_2_) [31], the more the olivines reduce the cracks during reduction, which also helps to improve the RDI. RI is mainly associated with chemical composition, particle size, porosity, mineral composition, and microstructure of the sinter. This is probably due to the variation of the mineral composition and microstructure as well as the porosity of the sinter, caused by the altering of the temperature and atmosphere through different levels of fuel combustion. The descending order of mineral reducibility is: hematite→calcium ferrite→magnetite→fayalite→iron silicate. It is easily found from Figure 5 and Figure 6 that with increasing fuel level, the fractions of hematite, fayalite, and FeOx with lower reducibility increase while hematite and calcium ferrite with higher reducibility decrease, hence the decease of RI. In addition, the reduction in pores (Figure 6) may be the other reason for the decrease of RI. On the other hand, with increasing fuel level, FeO increases (Table 4), and the more FeO the lower the RI, as the content of FeO is calculated through Fe^2+^ and the minerals that contain Fe^2+^ mostly contain the minerals with lower RI, such as Fe_3_O_4_, 2FeO·SiO_2_, CaO·FeO·SiO_2_, MgO·FeO·SiO_2_, and FeO.

Figure 10 shows that as the fuel level increases, the softening zone of the sinter shifts upwards and the softening interval becomes wider. The softening temperature of sinter in the heating up process mainly depends on the type and quantity of low-melting minerals. With lower formation temperature and higher quantities of low-melting minerals, the sinter softening temperature decreases. In contrast, the softening temperature is much higher. As can be seen in Table 4 and Figure 6, with increases in fuel level, FeO increases and low-melting minerals, such as fayalite, increase, which is also confirmed from Figure 5 and Figure 6. In the process of softening, there are two destinations of FeO in the sinter. One is reduced to metallic iron (Fe), the other is combined with gangue and forms the low-melting liquid. For the increase of FeO in sinter with the increasing fuel level, the FeO in the slag increases and more FeO combines with gangue to form more low-melting melt. For this reason, the softening temperatures decrease as the softening zone widens and the softening properties worsen.

In general, too low a fuel level will lead to too low a temperature in the sintered material layer and insufficient liquid phase, and too high a combustion level will lead to over-melting of the sintered ore and at the same time cause a lower oxygen potential in the sintered material layer, which is not conducive to the generation of calcium ferrate or to its decomposition, thus reducing the quality of the sintered minerals. This process is consistent with the effect of fuel level on Panzhihua vanadiferous titanomagnetite. While it can be observed that the sintering rate of RVT is greater than that of Panzhihua vanadiferous titanomagnetite, RDI is better than Panzhihua vanadiferous titanomagnetite sinter, but the RI of Panzhihua vanadiferous titanomagnetite sinter is better than that of RVT [27]. At the same time, a comprehensive evaluation of the production of RVT sinter ore at different fuel levels was carried out using the composite index method. The results showed that the composite index was the highest and the production was optimal at a fuel level of 4.0% coke powder ratio under the current production process and raw material conditions. This result is comparable to the fuel levels of domestic companies using vanadium and titanium ore smelting. Therefore, from the perspective of the application of sinter ore fuel, sintering using RVT can produce a qualified sinter ore that can be used for blast furnace smelting.

## 5. Conclusions

The influence of fuel level on RVT sinter properties, productivity, and mineralogy were researched by sintering pot tests, metallographic microscope, and SEM-EDS analysis, while the comprehensive index was evaluated through the comprehensive index method. Some conclusions were revealed as follows:

- Changes in temperature and atmosphere in the combustion zone caused by fuel combustion in turn affect the sintering process operation parameters. By increasing the fuel level from 3.5% to 4.0%, the flame front speed, TI, yield and productivity increase and then decrease, with the highest flame front speed and TI at 4% fuel level, the highest yield at 5.5% fuel level, and the highest productivity at 4.5% fuel level.
- The different fuel levels in the sinter mix affect the type and quantity of minerals in the sinter, as well as the microstructure of the minerals and thus the metallurgical properties of the sinter. With the increase in fuel level (3.5−6.0%), RDI_+3.15_ increased from 52.69% to 93.19%, RI decreased from 76.52% to 50.35%, the softening zone shifted, and the softening zone ΔT widened from 108.5 °C to 114 °C.
- With the increase in fuel level (3.5−6.0%), the contents of FeO, SiO_2_, and MgO increased, while TiO_2_ had a drop. Meanwhile, the number of pores and calcium ferrite and hematite decreased but had an increase in silicate (glassy, dicalcium silicate, olivine, etc.). In addition, in the fuel level ranged from 3.5% to 5.5% and magnetite content increased as fuel level gradually increased, but then had a drop. Moreover, when the fuel level increased to greater than 5.0%, FeOx and fayalite increased quickly and a small amount of metallic iron appeared under the fuel level of 6.0%.
- The comprehensive index of the effect of fuel level on RVT sinter properties and productivity was evaluated through the comprehensive index method, combined with the indicators and significance coefficient of PANGGANG. The optimal fuel level was determined to be 4.0% under current production conditions and indicators for the highest value of the comprehensive index.

## Figures and Tables

**Table 1:** Chemical composition of raw materials (mass, %).

Raw Materials	TFe	FeO	CaO	SiO_2_	MgO	Al_2_O_3_	TiO_2_	V_2_O_5_	Cr_2_O_3_	H_2_O
RVT ore	61.54	28.51	0.32	2.37	1.21	2.89	5.17	1.01	0.50	1.01
Magnetite A	62.87	25.79	0.46	5.16	1.03	3.40				0.27
Magnetite B	64.38	26.86	0.05	5.38	0.18	3.64				0
Magnetite C	61.85	23.11	1.36	3.36	3.44	2.45				0
Shaft furnace dust	62.47		0.33	8.46	0.61	0.93				0
Magnesite	0		1.21	3.55	42.24	0				2.01
Quicklime	0		80.1	5.02	1.10	0				0

**Table 2:** Industrial analysis of coke breeze and chemical composition of the ash (mass, %).

Fixed Carbon	Total Sulfur	Volatile	Ash (14.00)						∑
			FeO	CaO	SiO_2_	MgO	Al_2_O_3_	Others	
84.98	0.48	1.49	0.14	0.5	7.52	0.14	2.77	2.93	100.00

**Table 3:** Parameters of the sintering pot test.

Item	Parameter	Item	Parameter	Item	Parameter
Sinter bed depth	500 mm	Sintering pot diameter	150 mm	Granulation time	12 min
Ignition vacuum	5.0 kPa	Sintering vacuum	10.0 kPa	Ignition time	2 min
Ignition temperature	1100 °C	Moisture	7.5 ± 0.3%	Fuel level in blend mix	3.5–6.0%
Return fines content	14.0%	Height of hearth layer	20 mm	Basicity(R = CaO/SiO_2_)	2.25

**Table 4:** Chemical compositions of RVT sinters (+5 mm) at different fuel levels (mass, %).

Item	TFe	FeO	CaO	SiO_2_	MgO	Al_2_O_3_	TiO_2_	V_2_O_5_	Cr_2_O_3_
NO.1 Fuel-3.5%	54.46	6.86	12.10	5.37	1.65	2.96	1.47	0.279	0.108
NO.2 Fuel-4.0%	54.71	8.46	12.62	5.71	1.66	3.01	1.49	0.269	0.115
NO.3 Fuel-4.5%	54.85	9.26	12.08	5.77	1.65	3.01	1.48	0.275	0.114
NO.4 Fuel-5.0%	54.61	10.56	12.23	5.79	1.68	2.97	1.44	0.285	0.110
NO.5 Fuel-5.5%	55.23	12.26	12.50	5.88	1.69	3.01	1.42	0.264	0.102
NO.6 Fuel-6.0%	54.89	18.14	12.42	5.81	1.72	3.02	1.42	0.273	0.104

**Table 5:** Evaluation for the effects of fuel on RVT sinter by comprehensive index.

Items	Zi_1_	Zi_2_	Zi_3_	Zi_4_	Zi_5_	$\begin{array}{c} fi\;=\;\sum_{j=1}^{m}\omega jzij\\ (i\;=\;1,2,\ldots n;\;j\;=\;1,2,\ldots m;) \end{array}$	Fi = fi − f1 + 100
NO.1 Fuel = 3.5%	1.359	60.2	52.69	76.52	3.5	296.60	100.00
NO.2 Fuel = 4.0%	1.939	66.6	57.03	74.95	4.0	335.55	138.95
NO.3 Fuel = 4.5%	1.965	59.5	67.33	70.84	4.5	312.21	115.61
NO.4 Fuel = 5.0%	1.904	58.4	81.96	65.23	5.0	307.51	110.91
NO.5 Fuel = 5.5%	1.792	58.0	85.74	59.32	5.5	297.94	101.34
NO.6 Fuel = 6.0%	1.684	57.7	93.19	50.35	6.0	288.91	92.31
$\begin{array}{c} Rj=(zij)\max\\ -(zij)\min \end{array}$	0.606	8.9	40.5	26.17	2.5	Result: NO.2 > NO.3 > NO.4 > NO.5 > NO.1 > NO.6	
Wj	20	30	20	15	15		
$\omega j=\frac{Wj}{Rj}$	33.00	3.37	0.494	0.573	6		

## Data Availability

The data presented in this study are available on request from the corresponding author.
